# Burden of Multiple Chronic Conditions in Delaware, 2011–2014

**DOI:** 10.5888/pcd13.160264

**Published:** 2016-11-23

**Authors:** Sangeeta Gupta

**Affiliations:** 1 Department of Public and Allied Health Sciences, Delaware State University, Dover, Delaware.

## Abstract

The objective of this study was to use data from the Behavioral Risk Factor Surveillance System (BRFSS) to examine the prevalence of multiple chronic conditions (MCC) by select sociodemographic groups and determine the prevalence of most common MCC dyads and triads among Delaware adults. Combined data for 2011 through 2014 from BRFSS (n = 18,052) were analyzed to determine prevalence of MCC. Delaware adults were categorized as having 0, 1, 2, or 3 or more of the following diagnosed chronic conditions: angina, arthritis, asthma, cancer, chronic kidney disease, chronic obstructive pulmonary disease, diabetes, high blood pressure, high cholesterol, myocardial infarction (heart attack), obesity, or stroke. More than 65% of Delaware adults had at least 1 of the 12 selected chronic conditions. Furthermore, 36.8% of Delaware adults had MCC. The arthritis/obesity dyad and the arthritis/high blood pressure/high cholesterol triad were the 2 most prevalent MCC combinations. The findings of this study contribute information to the field of MCC research.

## Introduction

Chronic conditions, which affect millions of Americans every year, largely spring from a small set of common risk factors such as smoking, physical inactivity, unhealthy diet, and excessive alcohol consumption (1). Because of this interrelatedness, more and more people are living with not just 1 chronic condition but with 2 or more conditions (2,3). By definition, chronic conditions are conditions that last a year or more and require ongoing medical attention and/or limit activities of daily living (3). They include both physical conditions and mental disorders. Multiple chronic conditions (MCC) are defined as 2 or more chronic conditions that affect a person at the same time (1–3). Aging is a major nonmodifiable risk factor for most chronic conditions (3). As the US population ages and life expectancy increases, the number of people with MCC is expected to rise (2,3). People with MCC have a greater risk of dying prematurely and have increased hospitalizations and activity limitations (2,3). The prevalence of MCC needs to be monitored over time to understand patterns of disease and identify constellations of conditions that are most prevalent (4). Identifying the most common patterns of MCC can support public health professionals and health care providers in coordinating and managing care for various subgroups (3,4).

In 2010, the US Department of Health and Human Services (HHS) provided a strategic framework to improve health care outcomes of people with MCC (4). The framework proposed a paradigm shift within the context of how chronic illnesses are addressed in the United States — from an approach focused on individual chronic diseases to one that uses a MCC approach (4). Goal 4 of the framework emphasizes the importance of research to understand the epidemiology of MCC. Objectives of the framework include research stimulation to elucidate differences in MCC among various sociodemographic groups. Additional research is needed to identify the most common MCC dyads and triads (3,4). Research findings would fill knowledge gaps on MCC and assist public health programs in addressing the health care needs of the population with MCC (4,5).

MCC research has been increasing. In October 2012, the Centers for Medicare and Medicaid Services reported the prevalence of MCC among Medicare beneficiaries (6). Two of 3 Medicare beneficiaries were found to have MCC, accounting for 93% of total Medicare expenditures (6). In another study, National Health Interview Survey (NHIS) data were analyzed to examine the prevalence of MCC by selected sociodemographic groups; these data showed a MCC prevalence of 26% among adults (7).

Bolstering MCC research efforts at the state and local level will further strengthen the strategic framework proposed by HHS. Local data will support improved characterization of the MCC population and facilitate state health care providers and regional policy makers in coordinating and managing care for people with MCC (4–7).

This article presents findings on MCC in Delaware using Behavioral Risk Factor Surveillance System (BRFSS) data. The main objective of this study was to use data from BRFSS to examine the prevalence of MCC by selected sociodemographic groups and determine the prevalence of most common MCC dyads and triads among Delaware adults.

## Analysis

### Data source

BRFSS is a cross-sectional telephone survey of the civilian, noninstitutionalized adult population aged 18 years or older, conducted by the Centers for Disease Control and Prevention and state health departments. Established in 1984 in 15 states, BRFSS now collects data on health-related risk behaviors, chronic health conditions, and use of preventive services in all 50 states as well as the District of Columbia and 3 US territories (8). BRFSS completes more than 400,000 adult interviews each year, making it the largest continuously conducted health survey system in the world. The BRFSS questionnaire is designed to include a core set of questions used by all states and an additional set sponsored by each state that may be derived from optional modules developed by the Centers for Disease Control and Prevention or other appropriate sources. By collecting behavioral health risk data at the state and local level, BRFSS has become a powerful data source for chronic disease surveillance (8).

Data from 18,052 Delaware adults surveyed during 2011 through 2014 combined were analyzed. BRFSS response rates for Delaware varied from 37.4% in 2012 to 46.4% in 2014.

### Definitions

The HHS Interagency Workgroup on MCC and Office of the Assistant Secretary for Health generated a conceptual model to define chronic conditions in the United States and provide a framework for more consistent application of selected conditions to multiple data systems (9). The 20 chronic conditions are hypertension, congestive heart failure, coronary artery disease (CAD), cardiac arrhythmias, hyperlipidemia, stroke, arthritis, asthma, autism spectrum disorder, cancer, chronic kidney disease, chronic obstructive pulmonary disease (COPD), dementia, depression, diabetes, hepatitis, human immunodeficiency virus, osteoporosis, schizophrenia, and substance use disorders. The data systems used for defining these 20 MCC do not include BRFSS. In addition, studies on MCC use various numbers and combinations of the 20 recommended conditions, with selections determined by data availability. For example, studies by the Centers for Medicare and Medicaid Services used 19 of the recommended 20 conditions, whereas the NHIS used 10 conditions (6–9).

My analyses used 12 chronic conditions: angina, arthritis, asthma, cancer, chronic kidney disease, chronic obstructive pulmonary disease, diabetes, high blood pressure, high cholesterol, myocardial infarction (heart attack), obesity, and stroke. CAD is listed as one chronic condition by the HHS Workgroup, but data collection systems differ in reporting this condition as coronary heart disease, ischemic heart disease, or other terms. Angina and heart attack (myocardial infarction) are included in the category of CAD. BRFSS assesses CAD with 2 questions, 1 for angina and 1 for heart attack. Both variables were included in my analyses to assess CAD.

A 12th condition, obesity, which is not included in the recommended 20 conditions, was included in my analyses. Obesity has been identified as a chronic condition or disease in the scientific literature (10). More than one-third of US adults are obese. Obesity-related chronic conditions include heart disease, stroke, type 2 diabetes, high blood pressure, high cholesterol, and certain types of cancer. In June 2013, the American Medical Association officially recognized obesity as a disease (11).

Participants were identified as having a diagnosed condition if they had ever been told by a doctor or other health care provider that they had high blood pressure, high cholesterol, coronary heart disease (angina), stroke, diabetes, cancer, kidney disease, COPD, asthma, arthritis, or heart attack (myocardial infarction). BRFSS captures data only on conditions that were confirmed by a doctor or health professional, potentially leading to the underreporting of conditions that were undiagnosed or were not recalled by the respondent during the BRFSS interview.

For obesity, data on self-reported weight and height was used to calculate body mass index (BMI) (kg/m^2^). Participants were classified as obese if BMI was 30 or higher. Health insurance categories were based on a hierarchy of mutually exclusive categories, which were private coverage, public coverage (Medicaid, Medicare, others state programs), other coverage (TRICARE [formerly CHAMPUS], Veterans Affairs, or military health plans; and any other), and uninsured.

I counted the presence of the 12 selected chronic conditions and combined them into 4 mutually exclusive categories: 0, 1, 2, and 3 or more chronic conditions. I also generated weighted prevalences for the 10 most common MCC dyad and triad combinations. The 5 most common MCC dyad and triad combinations were further described by sex, age group, and county. These MCC dyad and triad combinations were also mutually exclusive.

### Statistical analyses

SAS version 9.3 (SAS Institute, Inc) was used to account for the complex sample design of BRFSS when generating estimates and confidence intervals. Descriptive estimates were calculated for subgroups defined by age, sex, race/ethnicity, and health insurance coverage. Two-tailed tests were used to test for significant differences in prevalence between population subgroups (*P* < .05). Three standard BRFSS data suppression guidelines were followed for the reporting of results: where 1) the numerator was less than 5, 2) the denominator was less than 50, and 3) the confidence interval was more than 20 points wide. If any of these guidelines were met for a given cell, the results for that cell were suppressed.

## Prevalence of MCC by Selected Sociodemographic Groups

During 2011 through 2014, more than 65% of Delaware adults had at least 1 of the 12 selected chronic conditions (Table 1); 36.8% had MCC, with 16.5% having 2 chronic conditions and 20.3% having 3 or more chronic conditions. The prevalence of MCC varied by sociodemographic group (Table 1). Women were more likely than men to have exactly 2 chronic conditions (*P* = .04) or 3 or more chronic conditions (*P* = .03). The prevalence of MCC was higher among non-Hispanic white adults than Hispanic adults among adults with 2 chronic conditions (*P* = .04). Among adults with 3 or more chronic conditions MCC prevalence was higher both among non-Hispanic whites and non-Hispanic blacks than among Hispanics (*P* = .001). The percentage of adults with MCC (both 2 and 3 or more) increased with age and was higher among those aged 65 years or older (*P* < .001). Differences in the prevalence of MCC (3 or more) were also found among the 3 counties in Delaware. Kent and Sussex counties had a higher prevalence than New Castle had (*P* < .001). Examination by health insurance coverage status indicated that MCC prevalence was greater among adults with public coverage than among adults with private or some other type of coverage.

**Table 1:** Prevalence of Chronic Conditions^a^ Among Delaware Adults by Selected Characteristics, Behavioral Risk Factor Surveillance System, 2011–2014

Characteristic	0 Chronic Conditions, % (95% CI)	1 Chronic Condition, % (95% CI)	2 Chronic Conditions, % (95% CI)	≥3 Chronic Conditions, % (95% CI)
**Total**	34.7 (33.6–35.8)	28.5 (27.5–29.5)	16.5 (15.7–17.2)	20.3 (19.6–21.1)
**Sex**				
Male	36.3 (34.6–38.0)	28.6 (27.0–30.2)	15.7 (14.6–16.8)	19.5 (18.3–20.6)
Female	33.3 (31.9–34.6)	28.4 (27.2–29.7)	17.2 (16.2−18.1)	21.1 (20.1–22.1)
**Race/ethnicity**				
White	33.4 (31.6–35.1)	28.9 (27.3–30.6)	17.5 (16.3–18.7)	20.2 (19.0–21.5)
Black	34.7 (30.8–38.7)	26.6 (23.0–30.2)	17.1 (14.2–19.8)	21.7 (18.7–24.6)
Hispanic	55.4 (46.6–64.1)	33.4 (24.6–42.2)	10.7 (6.30–15.1)	11.3 (7.1–15.4)
**Age, y**				
<65	40.3 (39.1–41.6)	29.9 (28.7–31.1)	14.9 (14.1–15.8)	14.9 (14.1–15.6)
≥65	11.9 (10.7−13.1)	22.9 (21.6–24.3)	22.6 (21.2–24.0)	42.6 (41.0–44.2)
**County**				
Kent	31.1 (29.0–32.9)	29.4 (27.5–31.3)	16.9 (15.6–18.4)	22.6 (21.1–24.2)
New Castle	37.1 (35.6–38.7)	29.1 (27.6–30.5)	15.9 (14.9–17.0)	17.9 (16.8–18.9)
Sussex	29.3 (27.4–31.2)	26.6 (24.8–28.4)	18.2 (16.8–19.5)	25.9 (24.4–27.5)
**Insurance**				
Public	22.7 (19.4–26.0)	30.5 (27.1–33.9)	22.1 (19.2–25.0)	24.7 (22.0–27.5)
Private	48.1 (45.0–51.3)	31.9 (28.9–34.9)	12.2 (10.6–13.9)	7.7 (6.4–9.1)
Other	37.9 (28.8–47.2)	26.1 (19.0–33.2)	17.7 (11.1–24.4)	18.2 (12.4–24.1)
Uninsured	33.4 (32.2–34.6)	27.8 (26.6–28.9)	16.7 (15.8–17.5)	22.2 (21.3–23.1)

Abbreviation: CI, confidence interval.

a Angina, arthritis, asthma, cancer, chronic kidney disease, chronic obstructive pulmonary disease, diabetes, high blood pressure, high cholesterol, myocardial infarction (heart attack), obesity, and stroke.

## Prevalence of MCC Dyads and Triads

For Delaware adults with 2 chronic conditions, the MCC dyad with the highest prevalence was arthritis and obesity (Table 2). This dyad was also the most common in both sexes, in all 3 counties, and in both age groups. The second most prevalent dyad was high blood pressure and high cholesterol. Table 2 lists the 5 most common dyads by sex, age, and county.

**Table 2:** Most Prevalent Chronic Condition^a^ Dyads by Age, Sex, and County for Delaware Adults with 2 Chronic Conditions, Behavioral Risk Factor Surveillance System, 2011–2014

Dyad	% (95% Confidence Interval)
**Total**	
Arthritis/obesity	15.6 (14.0–17.3)
High blood pressure/high cholesterol	9.1 (7.9–10.3)
Asthma/obesity	8.3 (6.8–9.8)
High blood pressure/obesity	6.5 (5.3–7.6)
Diabetes/obesity	5.4 (4.2–6.6)
Arthritis/asthma	5.3 (4.3–6.4)
Arthritis/high blood pressure	5.2 (4.2–6.2)
Obesity/high cholesterol	4.9 (4.0–5.9)
Arthritis/high cholesterol	4.8 (4.0–5.6)
Arthritis/cancer	3.4 (2.7–4.1)
**Sex**	
**Male**	
Arthritis/obesity	14.8 (12.1–17.5)
High blood pressure/high cholesterol	10.5 (8.4–12.6)
Asthma/obesity	9.4 (6.8–12.0)
Diabetes/obesity	7.2 (5.0–9.4)
High blood pressure/obesity	6.9 (5.1–8.6)
**Female**	
Arthritis/obesity	16.4 (14.2–18.5)
High blood pressure/high cholesterol	7.9 (6.6–9.2)
Asthma/obesity	7.4 (5.7–9.1)
Arthritis/asthma	7.2 (5.6–8.8)
High blood pressure/obesity	6.2 (4.7–7.6)
**Age, y**	
**<65**	
Arthritis/obesity	16.7 (14.5–18.8)
Asthma/obesity	11.1 (9.1–13.2)
High blood pressure/high cholesterol	8.4 (6.9–9.8)
High blood pressure/obesity	7.8 (6.3–9.3)
Diabetes/obesity	6.4 (4.8–8.0)
**≥65**	
Arthritis/obesity	12.9 (10.6–15.2)
High blood pressure/high cholesterol	11.1 (9.2–13.0)
Arthritis/cancer	7.01 (5.3–8.7)
Arthritis/high blood pressure	6.8 (5.2–8.4)
Arthritis/diabetes	6.2 (4.0−8.4)
**County**	
**Kent**	
Arthritis/obesity	16.1 (13.2–19.1)
Asthma/obesity	9.4 (6.5–12.2)
High blood pressure/obesity	8.8 (5.8–11.9)
High blood pressure/high cholesterol	8.3 (5.6–10.9)
Arthritis/asthma	6.5 (4.2–8.8)
**New Castle**	
Arthritis/obesity	15.3 (12.8–17.8)
High blood pressure/high cholesterol	9.4 (7.7–11.2)
Asthma/obesity	8.3 (6.0–10.6)
High blood pressure/obesity	5.8 (4.3–7.3)
Arthritis/high blood pressure	5.5 (4.0–7.1)
**Sussex**	
Arthritis/obesity	15.4 (12.4–18.4)
High blood pressure/high cholesterol	9.2 (7.3–11.1)
Asthma/obesity	8.1 (5.7–10.3)
High blood pressure/obesity	6.7 (4.6−8.8)
Arthritis/high cholesterol	6.5 (4.7–8.2)

a Angina, arthritis, asthma, cancer, chronic kidney disease, chronic obstructive pulmonary disease, diabetes, high blood pressure, high cholesterol, myocardial infarction (heart attack), obesity, and stroke.

Among Delaware adults with 3 or more chronic conditions, the MCC triad of arthritis, high blood pressure, and high cholesterol was the most prevalent (Table 3). This was also the most common triad in men, those aged 65 or older, and those in New Castle and Sussex counties. Arthritis, asthma, and obesity was the most common triad in women, in Kent County, and in those aged less than 65 years. Table 3 lists the 5 most common triads by sex, age, and county among Delaware adults with 3 or more chronic conditions.

**Table 3:** Most Prevalent Chronic Condition^a^ Triads by Age, Sex and County for Delaware Adults with 3 or More Chronic Conditions, Behavioral Risk Factor Surveillance System, 2011–2014

Triad	% (95% Confidence Interval)
**Total**	
Arthritis/high blood pressure/high cholesterol	7.7 (6.7–8.7)
Arthritis/asthma/obesity	7.2 (6.1–8.3)
Arthritis/diabetes/obesity	5.2 (4.4–6.1)
Arthritis/diabetes/high cholesterol	4.8 (4.1–5.5)
Arthritis/asthma/high cholesterol	4.2 (3.5–4.9)
High blood pressure/obesity/high cholesterol	4.1 (3.3–5.0)
Angina/heart attack/high cholesterol	3.1 (2.5–3.6)
Diabetes/high blood pressure/high cholesterol	3.1 (2.3–3.8)
Angina/heart attack/obesity	3.1 (2.3–3.8)
Arthritis/cancer/obesity	2.1 (1.5–2.7)
**Sex**	
**Male**	
Arthritis/high blood pressure/high cholesterol	7.5 (5.8–9.2)
High blood pressure/obesity/high cholesterol	5.7 (4.2–7.3)
Arthritis/diabetes/obesity	5.1 (3.8–6.2)
Arthritis/diabetes/high cholesterol	4.7 (3.5–5.9)
Angina/heart attack/obesity	4.5 (3.0–6.0)
**Female**	
Arthritis/asthma/obesity	9.9 (8.1–11.6)
Arthritis/high blood pressure/high cholesterol	7.9 (6.7–9.1)
Arthritis/asthma/high cholesterol	6.6 (5.4–7.7)
Arthritis/diabetes/obesity	5.4 (4.2–6.6)
Arthritis/diabetes/high cholesterol	4.9 (4.0–5.8)
**Age, y**	
**<65**	
Arthritis/asthma/obesity	10.1 (8.3–11.8)
High blood pressure/obesity/high cholesterol	6.5 (5.1–7.9)
Arthritis/high blood pressure/high cholesterol	6.3 (4.9–7.7)
Arthritis/diabetes/obesity	5.1 (3.8–6.3)
Arthritis/diabetes/high cholesterol	4.5 (3.5–5.5)
**≥65**	
Arthritis/high blood pressure/high cholesterol	9.7 (8.3–11.2)
Arthritis/diabetes/obesity	5.5 (4.4–6.6)
Arthritis/diabetes/high cholesterol	5.2 (4.2–6.2)
Angina/heart attack/high cholesterol	4.9 (3.8–6.0)
Angina/heart attack/obesity	3.9 (2.5–5.2)
**County**	
**Kent**	
Arthritis/asthma/obesity	9.9 (7.5–12.3)
Arthritis/diabetes/obesity	6.4 (4.5–8.2)
Arthritis/high blood pressure/high cholesterol	5.7 (4.3–7.1)
Arthritis/diabetes/high cholesterol	5.6 (4.2–7.0)
Arthritis/asthma/high cholesterol	4.8 (3.3–6.2)
**New Castle**	
Arthritis/high blood pressure/high cholesterol	8.5 (6.8–10.2)
Arthritis/asthma/obesity	6.9 (5.4–8.6)
Arthritis/diabetes/obesity	5.4 (4.0–6.8)
High blood pressure/obesity/high cholesterol	4.6 (3.3–6.0)
Arthritis/asthma/high cholesterol	4.4 (3.3–5.5)
**Sussex**	
Arthritis/high blood pressure/high cholesterol	7.7 (6.2–9.2)
Arthritis/asthma/obesity	6.1 (4.3–7.8)
Arthritis/diabetes/high cholesterol	5.1 (3.7–6.3)
Arthritis/diabetes/obesity	4.2 (3.1–5.3)
Angina/heart attack/high cholesterol	3.9 (2.8–5.1)

a Angina, arthritis, asthma, cancer, chronic kidney disease, chronic obstructive pulmonary disease, diabetes, high blood pressure, high cholesterol, myocardial infarction (heart attack), obesity and stroke.

## Summary

Nearly 37% of Delaware adults have MCC, based on BRFSS data from 2011 through 2014. More Delaware adults had 3 or more chronic conditions (20.3%) compared with those with 2 chronic conditions (16.5%). Also, these MCC prevalence findings are considerably higher than those in the study using NHIS data (7) because of differences in the number and type of chronic conditions studied. Including obesity as one of the chronic conditions may have been the driving factor. Nearly 31% of Delaware adults were obese in 2014 (8). However, the general distribution of MCC among sociodemographic groups was similar to that found in previous research. Among certain subgroups (such as women and older adults), the prevalence of MCC was generally higher, and for others (Hispanic adults and those with private insurance) the prevalence was generally lower. These findings were consistent with those of earlier studies (6,7). The arthritis/obesity dyad and the arthritis/high blood pressure/high cholesterol triad were 2 of the most prevalent MCC combinations, differing from the most common MCC combinations found in the NHIS study (7).

This study has limitations. BRFSS is a telephone survey that excludes people living in institutions, nursing homes, long-term care facilities, and correctional institutions, and results might not be applicable to these populations. Second, BRFSS data are self-reported and subject to recall and social desirability bias (eg, underreporting of actual weight). Also, BRFSS captures data only on conditions that were confirmed by a doctor or health professional, potentially leading to the underreporting of conditions that were undiagnosed or were not recalled by the respondent during the BRFSS interview. In addition, BRFSS captures data on only 10 of 20 conditions listed in the HHS Interagency Workgroup definition (4,5). Not all mental health conditions in the list of 20 conditions are included in the BRFSS Core questionnaires and therefore were not included in these analyses. Because not all 20 conditions were included, these analyses most likely underestimate the burden of MCC.

Despite these limitations, examining the prevalence of MCC among subgroups of adults allowed the identification of MCC patterns in the Delaware adult population. Future studies could seek to explain why differences in the prevalence of MCC among subgroups exist. Strategies of the HHS MCC initiative (4,5) include stimulation of epidemiologic research to understand the patterns of MCC. The findings of this study contribute information to the field of MCC research. In addition, providing MCC data at the state and county level can improve targeting of interventions by caregivers and policy makers.
